# Genome-Wide Identification and Characterization of the *SMXL* Gene Family in *Lavandula angustifolia*

**DOI:** 10.3390/ijms27104461

**Published:** 2026-05-16

**Authors:** Yongguang Li, Kaihang Zhang, Xiaoru Zhang, Hongxuan Li, Hanyi Li, Bingbing Liu, Xiaoxing Wang, Chunqiao Wang, Yang Yang, Jiandong Ren, Cuijie Cui, Nuerkaimaier Mulati, Shangfu Ren, Bin Ma, Jishan Xiang

**Affiliations:** 1 Xinjiang Key Laboratory of Lavender Conservation and Utilization, College of Biological Sciences and Technology, Yili Normal University, Yining 835000, China; zongheng1476408@163.com (Y.L.); venchy790@163.com (K.Z.); xiaoruxj@163.com (X.Z.);; 2 College of Agriculture, Nanjing Agricultural University, Nanjing 210095, China; 3 College of Life and Geography, Kashi University, Kashi 844000, China

**Keywords:** strigolactones, *Lavandula angustifolia*, *SMXL* gene family, plant architecture, floral transition

## Abstract

SMXL proteins serve as central regulators of strigolactone (SL) and karrikin (KAR) signaling pathways, orchestrating key developmental processes including shoot branching, floral transition, photomorphogenesis and stress responses. However, the *SMXL* gene family has not been systematically characterized in *Lavandula angustifolia*. We identified 37 *LaSMXL* genes in the lavender genome. Phylogenetic and synteny analyses classified these proteins into four subgroups (Groups I–IV) and indicated that family expansion in lavender was mainly driven by whole-genome and segmental duplications, with most duplicated pairs evolving under strong purifying selection. Gene structure and motif analyses revealed high conservation within each subgroup. Promoter *cis*-element analysis suggested that *LaSMXL* genes are integrated into light-, hormone- and stress-responsive regulatory networks. RNA-seq profiling showed that most *LaSMXL* genes are weakly expressed, but a small subset displays pronounced tissue specificity and clear transcriptional responses to low temperature. Protein–protein interaction predictions and co-expression network analysis further placed highly expressed *LaSMXLs* within conserved SL/KAR and chloroplast/light-associated modules, alongside D14, KAI2, MAX2, CCD7/CCD8, and CYP711A. Together, these findings provide the first comprehensive overview of the *SMXL* gene family in lavender and identify candidate *LaSMXL* genes for future functional studies aimed at optimizing plant architecture and inflorescence-derived essential oil biosynthesis.

## 1. Introduction

Plant growth and development are orchestrated by multilayered signaling networks, in which phytohormones act as central endogenous regulators that rapidly integrate environmental cues [1]. Strigolactones (SLs), initially identified in root exudates as germination stimulants for parasitic weed seeds [2,3], have since been shown to regulate diverse developmental processes in higher plants, including shoot branching, leaf senescence, secondary wall formation, photomorphogenesis, and stem elongation [4,5]. Functionally analogous signals, karrikins (KARs) and karrikin-like (KL) compounds, are perceived by the KARRIKIN-INSENSITIVE2 (KAI2) receptor in association with the SKP1–Cullin–F-box MAX2 (SCF^MAX2^) complex, highlighting a structural and functional parallelism with SL signaling [6,7].

In higher plants, D53-like/SUPPRESSOR OF MAX2 1-LIKE (SMXL) proteins act as central downstream targets and key transcriptional regulators of plant architecture, perception by the SL receptor DWARF14 (D14), the F-box protein MORE AXILLARY GROWTH2/DWARF3 (MAX2/D3) is recruited to form the SCF^MAX2^ complex, which polyubiquitinates SMXL6/7/8 and targets them for 26S proteasome–mediated degradation, thereby releasing the transcription factor BRANCHED1 (BRC1) from repression and promoting axillary outgrowth [8,9,10]. KAR/KL signals act through the KAI2–SCF^MAX2^ module to degrade SMAX1/SMXL2, together constituting the conserved D14/KAI2–MAX2–SMXL signaling axis [11]. Recent evidence indicates that SMXL stability is not solely MAX2 dependent but is also influenced by environmental factors (light, temperature, nutrients) and additional ubiquitin ligases [12,13,14]. For instance, the CULLIN4–DWD HYPERSENSITIVE TO ABA1 (CUL4–DWA1) complex targets SMXL6/7/8 in *Arabidopsis*, while PROTEOLYSIS1 (PRT1) mediates degradation of MdSMXL8 in *Malus domestica* (apple), revealing alternative MAX2-independent mechanisms [15,16].

Moreover, SMXL proteins are increasingly recognized not merely as repressors but as signaling hubs integrating multiple cues. Through the conserved ETHYLENE RESPONSE FACTOR–associated amphiphilic repression (EAR) motif in their D2 domain, SMXL proteins recruit TOPLESS (TPL)/TPL-related co-repressors and chromatin remodelers; in parallel, they can bind DNA directly or interact with diverse transcription factors—including SPL, TCP, PIF, DELLA, WRKY, and BES1/BZR1—to modulate gene expression by altering TF binding, activity, or stability [13,17,18,19,20,21]. Notably, SL–AtD14 signaling was recently shown to delay flowering through dual mechanisms: direct binding and stabilization of the AP2 factor TARGET OF EARLY ACTIVATION TAGGED 1 (TOE1), and AtD14-mediated degradation of SMXL7, which liberates TOE1 to repress *FLOWERING LOCUS T* (*FT*) [22]. Consistently, SL-deficient biosynthetic or signaling mutants (*max3*, *Atd14*, *max2*) exhibit early flowering, whereas the *smxl6/7/8* triple mutant shows the opposite phenotype [9,23], underscoring the pivotal role of SMXL6/7/8 in the vegetative-to-reproductive transition. Collectively, SMXL proteins operate at the interface of receptor–E3 ligase–proteasome signaling and transcriptional/chromatin regulation, integrating hormonal and environmental inputs to fine-tune branching, flowering time, and stress responses, thus establishing a conceptual foundation for molecular crop improvement [24].

Importantly, lavender is cultivated primarily for its floral biomass. Its inflorescences are the main harvested organs and the primary sites of essential oil accumulation. Therefore, flower development dictates not only the number of harvestable spikes but also the abundance and function of secretory structures needed for essential oil biosynthesis. Consequently, branching architecture and flowering time directly impact spike density and the flowering period. Ultimately, these developmental traits determine both the yield and the compositional stability of the essential oil at harvest. Given that the SL–SMXL signaling pathway is a central regulatory axis governing axillary bud outgrowth, reproductive transition, and abiotic stress adaptation, it is well positioned to integrate environmental signals with developmental outputs in lavender. Therefore, this study aimed to systematically characterize the *LaSMXL* gene family at the genome-wide level. Specifically, we investigated their evolutionary dynamics and profiled their spatio-temporal expression patterns across diverse tissues and distinct floral developmental stages. Furthermore, we evaluated their transcriptional responses to low-temperature stress and mapped their interaction and co-expression networks. By integrating these evolutionary, expressional, and regulatory insights, this study aims to uncover key molecular switches controlling branching and flowering-related traits, ultimately providing candidate targets for optimizing lavender plant architecture and further improving essential oil yield.

## 2. Results

### 2.1. Identification and Chromosomal Distribution of LaSMXL Genes

Genome-wide screening of the *L. angustifolia* genome identified 37 putative *SMXL* genes, which were designated *LaSMXL1–LaSMXL37* according to their chromosomal locations (Table S1). The encoded proteins range in length from 559 amino acids (LaSMXL19) to 1094 amino acids (LaSMXL18, LaSMXL23). Analysis of physicochemical properties revealed considerable variation among LaSMXL members: the predicted molecular weights (MW) span from 61,442.02 Da (LaSMXL19) to 120,912.37 Da (LaSMXL23), whereas the theoretical isoelectric points (pI) range from 5.74 (LaSMXL27) to 8.85 (LaSMXL19). GRAVY index values vary between −0.482 (LaSMXL22) and −0.174 (LaSMXL21), indicating that all LaSMXL proteins are hydrophilic. Subcellular localization predicted by WoLF PSORT II suggests that the 37 LaSMXL proteins are distributed across three major cellular compartments: 24 proteins are predicted to localize to the nucleus and 10 to plastids, whereas the remaining three are predicted to localize either to both the cytoplasm and nucleus (LaSMXL17 and LaSMXL27) or to mitochondria (LaSMXL34).

The chromosomal distribution of the 37 *LaSMXL* genes was examined using TBtools (Figure S1). In total, 32 *LaSMXL* loci were anchored to 20 of the 27 chromosomes, whereas the remaining seven chromosomes contained no detectable *LaSMXL* genes. Among the chromosomes bearing *LaSMXL* loci, Chr3 and Chr7 each harbor four genes, Chr5 contains three genes, and Chr6, Chr8, Chr20, and Chr24 each carry two genes, while the other *LaSMXL*-bearing chromosomes each contain a single gene. The remaining five members, *LaSMXL1–LaSMXL5*, are located on five unplaced scaffolds.

**Figure 1:**
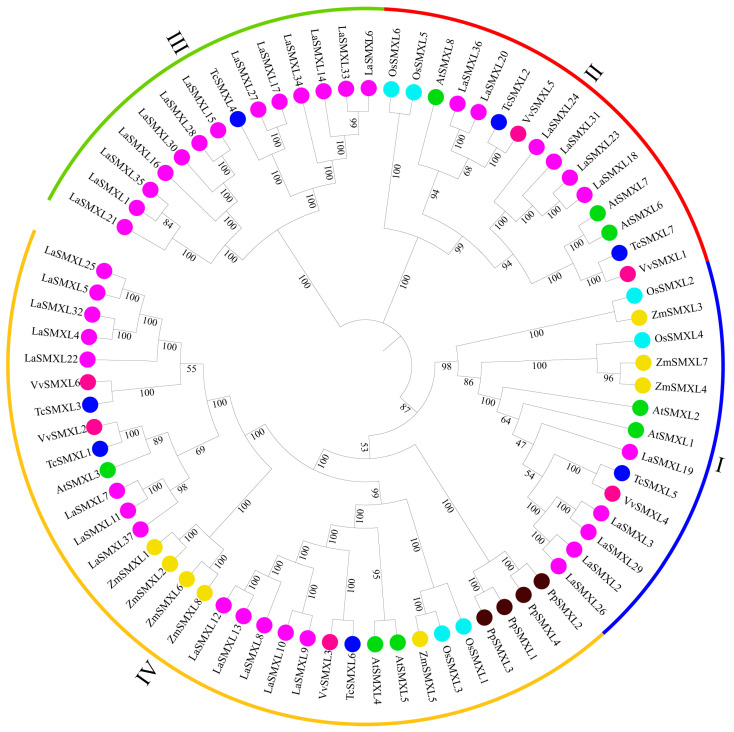
Phylogenetic analysis of SMXL proteins from *A. thaliana* (*At*), *O. sativa* (*Os*), *Z. mays* (*Zm*), *V. vinifera* (*Vv*), *P. patens* (*Pp*), *T. cacao* (*Tc*), and *L. angustifolia* (*La*). The phylogenetic tree was divided into four groups (Groups I–IV). LaSMXL proteins are marked with purple dots.

To elucidate the phylogenetic relationships among LaSMXL proteins, we constructed a phylogenetic tree using the full-length amino acid sequences of LaSMXLs together with SMXL proteins from *Arabidopsis thaliana*, *Oryza sativa*, *Zea mays*, *Vitis vinifera*, *Physcomitrium patens*, and *Theobroma cacao* (Figure 1). The LaSMXL proteins were resolved into four distinct groups (Groups I–IV), consistent with the classification reported for AtSMXL and GmSMXL proteins, suggesting that SMXL family diversification is relatively conserved across dicotyledonous and monocotyledonous plants. Group III comprises 13 LaSMXL proteins, including LaSMXL21, LaSMXL1, and LaSMXL35. Group II contains six LaSMXL proteins that cluster most closely with AtSMXL6, AtSMXL7, and AtSMXL8. Group I includes five LaSMXL proteins that group together with AtSMXL1 and AtSMXL2, indicating a close evolutionary relationship. Group IV is the largest clade in the lavender SMXL family and comprises 13 LaSMXL proteins, such as LaSMXL25, LaSMXL5, LaSMXL32, and LaSMXL4, which are phylogenetically closest to AtSMXL3, AtSMXL4, and AtSMXL5.

### 2.2. Gene Structure, Conserved Motifs, and Structural Diversification of LaSMXL Proteins

**Figure 2:**
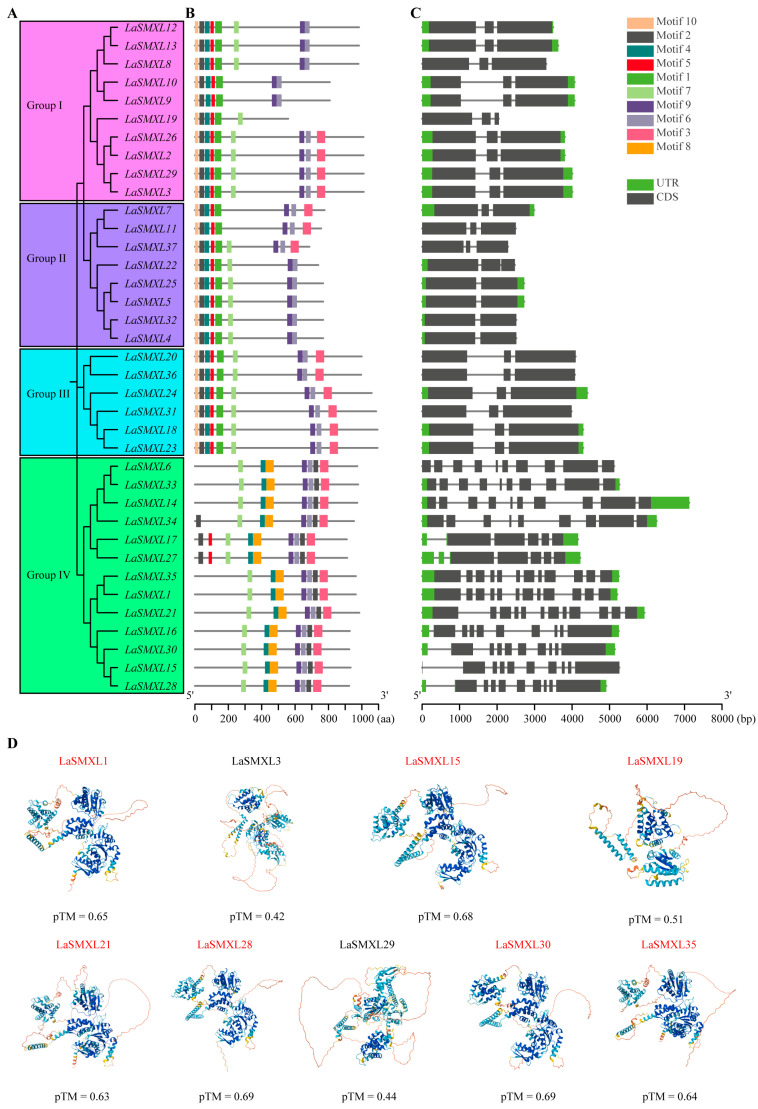
Phylogenetic relationships, conserved motifs, and gene structures of *LaSMXL* genes. (**A**) Phylogenetic tree of SMXL proteins in *L. angustifolia*. (**B**) Distribution of conserved motifs among the 37 LaSMXL proteins. (**C**) Exon–intron organization of the 37 *LaSMXL* genes. Coding exons and untranslated regions (UTRs) are represented by gray and green boxes, respectively. (**D**) Predicted three-dimensional structures of selected LaSMXL proteins. The three-dimensional structures of nine highly expressed LaSMXL proteins were predicted using AlphaFold 3 Models with predicted TM-scores (pTM) > 0.5 indicate reliable global structural topology; corresponding proteins are marked in red.

To systematically characterize the gene structures and conserved motifs of SMXL family members in lavender, we first constructed a phylogenetic tree of LaSMXL proteins and then performed motif and gene structure clustering analyses according to the resulting groupings (Figure 2A). Using the MEME online suite, we identified 10 conserved motifs among the 37 LaSMXL proteins (Figure 2B). Members within the same phylogenetic group generally exhibited highly similar motif compositions. Groups I–III showed comparable motif patterns, all containing motifs 10, 2, 4, 5, and 1. By contrast, motifs 7, 9, 6, and 3 were variably present: motif 7 was absent from LaSMXL10, LaSMXL9, LaSMXL7, and LaSMXL11, whereas motifs 9 and 6 were both missing from LaSMXL19. Motif 3 was only detected in a subset of genes, including LaSMXL26, LaSMXL2, LaSMXL29, LaSMXL3, LaSMXL7, LaSMXL11, LaSMXL37, LaSMXL20, LaSMXL36, LaSMXL24, LaSMXL31, LaSMXL18, and LaSMXL23. Group IV was characterized by a distinct motif combination comprising motifs 4, 7, 8, 9, 6, and 3. Compared with other Group IV members, LaSMXL17 and LaSMXL27 contained additional motifs 2 and 5, whereas LaSMXL34 contained an additional motif 2.

The exon–intron structures of *LaSMXL* genes were determined using the Gene Structure Display Server (GSDS 2.0) (Figure 2C). Most *LaSMXL* genes possessed both 5′ and 3′ UTRs, with the exception of *LaSMXL8*, *LaSMXL19*, *LaSMXL11*, *LaSMXL37*, *LaSMXL20*, *LaSMXL36*, and *LaSMXL6*, which lacked UTRs. The gene structures of Groups I and III were highly similar, typically comprising three exons and two introns. Group II showed a comparable overall organization, although the third exon was shorter, and *LaSMXL25*, *LaSMXL5*, *LaSMXL32*, and *LaSMXL4* each contained only two exons. Notably, the gene structure of *LaSMXL22* resembled that of other Group II members, but its second exon was split, resulting in a configuration with three exons and two introns. Group IV contained 13 genes whose structures were generally longer and more complex than those of the other groups, with exon numbers exceeding nine in most cases. *LaSMXL17* and *LaSMXL27* were exceptions within Group IV, each consisting of five exons and four introns.

To evaluate structural conservation among LaSMXL proteins, the three-dimensional (3D) structures of nine highly expressed members were predicted using AlphaFold 3 (Figure 2D). Seven proteins exhibited predicted models with pTM scores greater than 0.5, indicating reliable overall folds and high structural similarity, and clustered within Group I in the phylogenetic analysis. In contrast, three proteins showed lower pTM scores (<0.5) and were phylogenetically related to *Arabidopsis* AtSMXL1/2 homologs associated with karrikin signaling. These results indicate that LaSMXL proteins display both structural conservation and diversification, consistent with functional differentiation within the family.

### 2.3. Synteny and Evolutionary Analyses of LaSMXL Gene Members

**Figure 3:**
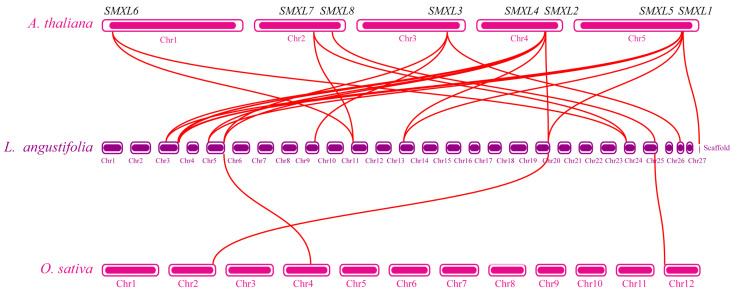
Syntenic analysis of *LaSMXL* genes between *L. angustifolia*, *A. thaliana*, and *O. sativa*. The red lines highlight homologous gene pairs between the two species.

The expansion and contraction of gene families is a key evolutionary process that enables plants to adapt to changing environments. To investigate the evolutionary dynamics of the SMXL family in lavender, we performed inter-genomic synteny analysis among *A. thaliana*, *O. sativa*, and *L. angustifolia* (Figure 3). Using the eight *Arabidopsis SMXL* genes as queries, we identified 14 syntenic *SMXL* genes in lavender. Among them, *AtSMXL1* was collinear with three lavender *SMXL* genes, *AtSMXL2* with two, *AtSMXL3* with three, *AtSMXL4* with five, *AtSMXL5* with five, *AtSMXL6* with two, *AtSMXL7* with two, and *AtSMXL8* with one, with some lavender genes serving as shared syntenic counterparts for multiple *Arabidopsis SMXLs*. These patterns indicate that the *SMXL* family has undergone substantial expansion in lavender, although the extent of expansion differs among individual *SMXL* orthologous groups.

Subsequent analysis of genomic collinearity between rice and lavender revealed that only 3 homologous genes of the 14 lavender *SMXL* genes are colinear with those in rice. This clearly indicates a distant kinship and early species divergence between lavender and rice. Further intra-genomic synteny analysis within the lavender genome identified 35 duplicated *LaSMXL* gene pairs (Figure S2). The connecting lines across different chromosomes represent these gene pairs, which exhibit a distinct pattern of segmental duplication. This indicates that segmental duplication events played a predominant role in the evolutionary expansion of the *LaSMXL* gene family within the lavender genome. Selection pressure analysis of these duplicated pairs showed that most *LaSMXL* genes have been subject to purifying selection (*K*a/*K*s < 1), with the notable exception of the *LaSMXL1*/*LaSMXL35* pair, which appears to have experienced positive selection (*K*a/*K*s > 1) (Table S2). Together, these results support a model in which whole-genome/segmental duplication followed by predominantly purifying selection—and limited adaptive divergence—has shaped the current LaSMXL repertoire in lavender while preserving key components of the SL/KAR signaling framework.

### 2.4. Cis-Element Analyses of LaSMXL Genes

To further elucidate the potential regulatory patterns and functions of *LaSMXL* genes, we used the PlantCARE web server to identify *cis*-acting elements within the 2000-bp upstream promoter regions of all *LaSMXL* loci. A broad spectrum of *cis*-elements associated with light responsiveness, growth and development, phytohormone signaling and stress responses was detected and grouped into three major categories: growth- and development-related, hormone-responsive and stress-responsive elements. Apart from ubiquitous light-responsive elements, *LaSMXL* promoters contained only a small number of additional growth- and development-related motifs (typically fewer than two per gene).

**Figure 4:**
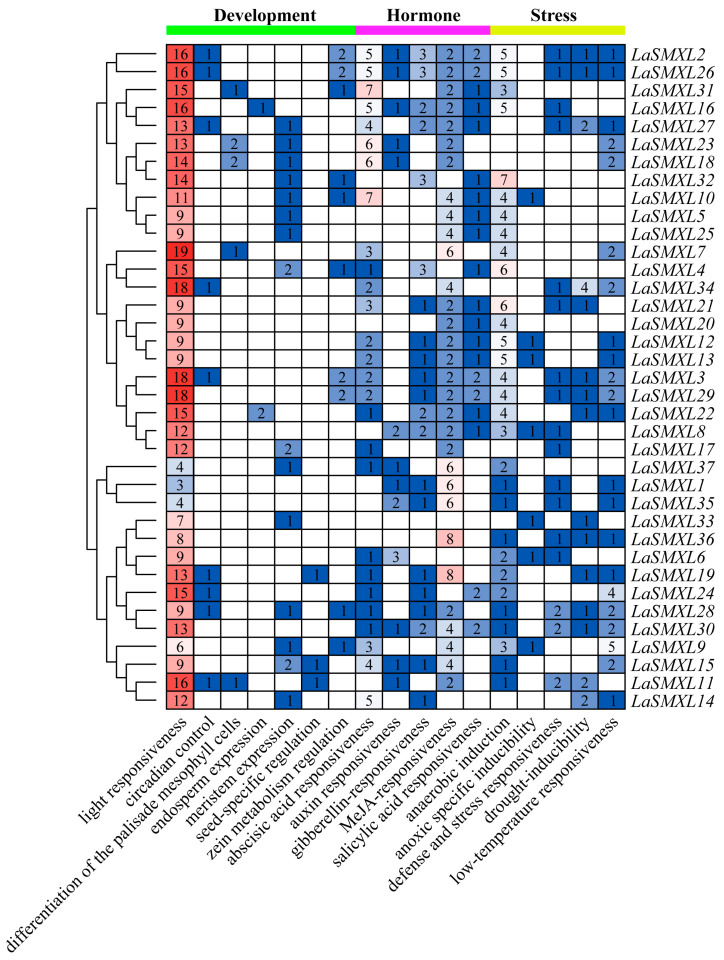
*Cis*-elements in the *LaSMXL* gene promoter regions. The number of *cis*-elements is shown in the heatmap, with darker red indicating a higher count. Numbers within cells represent the specific *cis*-element count for each gene.

Five classes of hormone-responsive *cis*-elements were identified, associated with MeJA (CGTCA-motif, TGACG-motif), salicylic acid (TCA-element), abscisic acid (ABRE), auxin (AuxRR-core, TGA-box), and gibberellin (GARE-motif, P-box). Among these, *LaSMXL31* and *LaSMXL10* harbored the highest numbers of ABA-responsive elements (seven each), whereas *LaSMXL36* and *LaSMXL19* contained the most MeJA-responsive elements (eight, respectively). Other hormone-related motifs occurred at low frequencies and were restricted to a subset of *LaSMXL* promoters (Figure 4).

In addition, five types of stress-responsive *cis*-elements were detected, including motifs associated with drought inducibility, low-temperature responsiveness, general defense and stress responses, anoxic-specific induction and anaerobic induction (Figure 4). Notably, anaerobic-induction elements were widely and abundantly distributed across *LaSMXL* promoters. Taken together, these *cis*-regulatory signatures indicate that *LaSMXL* gene expression is likely modulated by multiple hormonal cues and diverse environmental stresses, providing a regulatory basis for the tissue-specific and temperature-responsive expression patterns observed in lavender.

### 2.5. Expression Patterns of LaSMXL Genes in Lavender

**Figure 5:**
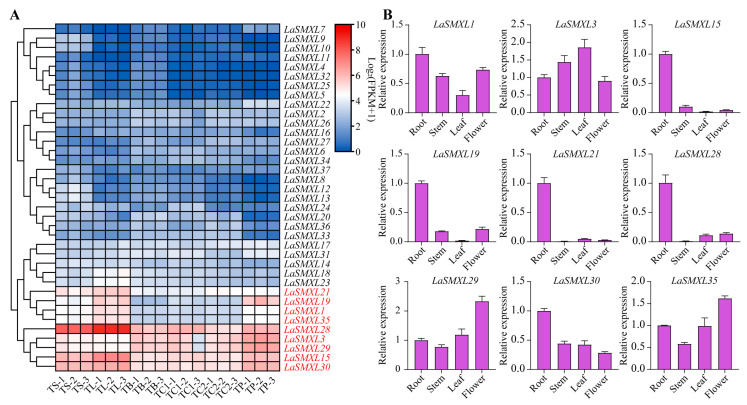
Expression characteristics of *LaSMXL* genes. (**A**) Expression heatmap of *LaSMXL* genes in different tissues of lavender. Genes highlighted in red font were selected for qPCR validation. TS, stem; TL, leaf; TB, bud; TP, petal; TC1, fresh calyx; TC2, mature calyx. (**B**) qPCR validation of the expression levels of selected *LaSMXL* genes. Error bars indicate mean ± SD (*n* = 3).

To investigate the potential functions of *SMXL* genes in lavender, we analyzed the transcriptomic expression profiles of *LaSMXL* genes across multiple tissues and developmental stages. The tissue-specific heatmap showed that most *LaSMXL* members were either not expressed or only weakly expressed in stems, leaves, flower buds, petals, and sepals (Figure 5A). Only a small subset of genes displayed pronounced tissue-enriched or broadly constitutive expression. Among these, *LaSMXL21*, *LaSMXL19*, *LaSMXL1*, and *LaSMXL35* were strongly expressed in stems, leaves and petals, but showed low transcript levels in flower buds, and sepals. *LaSMXL28*, *LaSMXL3*, *LaSMXL29*, *LaSMXL15*, and *LaSMXL30* were expressed in all sampled tissues, albeit with distinct preferences: *LaSMXL28* accumulated predominantly in stems and leaves, whereas *LaSMXL3* and *LaSMXL29* were more highly expressed in flower buds, sepals, and petals. *LaSMXL15* and *LaSMXL30* also exhibited elevated expression in stems, leaves, and petals.

**Figure 6:**
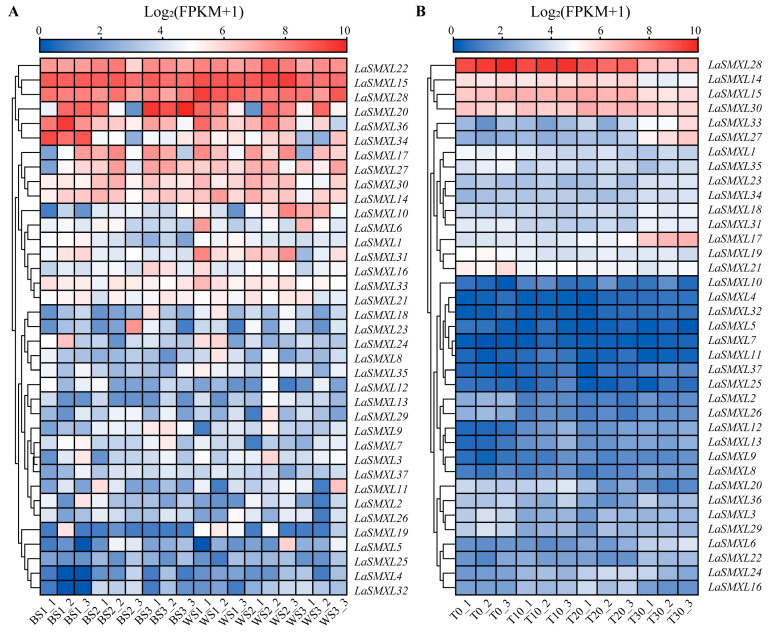
Expression patterns of the *LaSMXL* gene family during lavender floral development and under temperature treatments. (**A**) Expression profiles of the *SMXL* gene family in two lavender cultivars (*Xinxun 2* and *YXA-5*) across developmental stages. B indicates *Xinxun 2*, and W indicates *YXA-5*. S1, S2, and S3 correspond to Bud stage I, Bud stage II, and 50% flowering, respectively. (**B**) Expression patterns of *LaSMXL* genes in *Lavandula angustifolia* under different temperature treatments. T0, T10, T20, and T30 denote 0 °C, 10 °C, 20 °C, and 30 °C, respectively.

To elucidate the expression behaviors of the *LaSMXL* gene family during lavender floral organ development, we performed transcriptome analyses of *LaSMXL* genes in two cultivars, ‘*Xinxun 2*’ and ‘*YXA-5*’, at three key developmental stages: bud stage I, bud stage II, and 50% flowering. The results showed that the *LaSMXL* gene family displayed heterogeneous expression patterns across the examined stages (Figure 6A), with the majority exhibiting low or undetectable expression in the assessed floral developmental stages. Nevertheless, some *LaSMXL* genes showed distinct expression profiles and could be categorized into four groups according to their expression dynamics: the first group included *LaSMXL22*, *LaSMXL15*, and *LaSMXL28*, which sustained high expression across all three floral developmental stages in both cultivars; the second group, represented by *LaSMXL20*, *LaSMXL36*, and *LaSMXL34*, was highly expressed at early stages and subsequently decreased markedly at 50% flowering; the third group (*LaSMXL17*, *LaSMXL27*, *LaSMXL30*, and *LaSMXL14*) showed relatively constant expression across the three stages, with an overall transcript abundance lower than that of the consistently high-expression group I; and the fourth group included genes such as *LaSMXL10*, *LaSMXL6*, *LaSMXL1*, *LaSMXL31*, *LaSMXL16*, *LaSMXL33*, and *LaSMXL21*, which were detected at low levels only at specific stages and showed relatively low overall abundance. Collectively, the *LaSMXL* gene family showed stage-dependent and diversified expression profiles during lavender floral organ development.

RNA-seq profiling under different temperature treatments indicated that most *LaSMXL* genes showed only modest changes in transcript abundance (Figure 6B). However, several members displayed clear temperature-responsive patterns: *LaSMXL28* and *LaSMXL14* showed progressively increased expression as temperature decreased from 30 °C to 0 °C, whereas *LaSMXL33*, *LaSMXL27*, and *LaSMXL17* exhibited gradually reduced expression across the same gradient. By contrast, *LaSMXL15* and *LaSMXL30* maintained relatively stable expression levels under all temperature conditions, suggesting potential roles in basic growth processes rather than stress responses.

To validate the RNA-seq data, we performed qPCR analysis on nine highly expressed *LaSMXL* genes and additionally quantified their transcript levels in roots (Figure 5B). The qPCR profiles were largely consistent with the RNA-seq patterns and further demonstrated that these genes also exhibit strong expression in roots, supporting their potential involvement in both root and aerial tissue development and providing a robust foundation for subsequent functional characterization.

### 2.6. Protein–Protein Interaction Network

**Figure 7:**
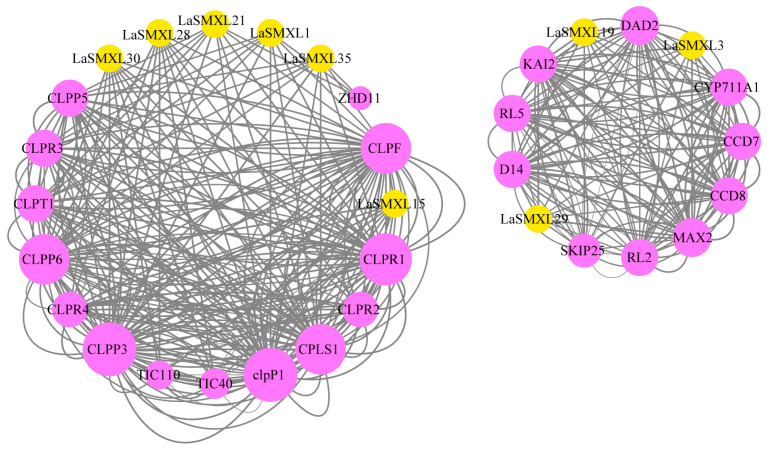
Protein–protein interaction network of LaSMXL proteins in lavender. Edge thickness increases with the combined_score value (0–1), indicating higher confidence for the predicted interaction between two proteins. Node size reflects the degree of connectivity. The yellow node represents the core LaSMXL protein in lavender, and the surrounding purple nodes represent its interacting partners.

To elucidate the potential functions of the nine highly expressed LaSMXL proteins, we predicted protein–protein interactions (PPIs) and constructed a STRING-based PPI network (Figure 7). The interaction partners of LaSMXL30, LaSMXL28, LaSMXL21, LaSMXL1, LaSMXL35, and LaSMXL15 largely overlapped and were comparable in number, whereas LaSMXL3, LaSMXL19, and LaSMXL29 each associated with a smaller set of ~10 proteins. Notably, the network recovered multiple canonical components of SL and KAR biosynthesis and signaling. These included the α/β-hydrolase receptors D14 and DAD2, which perceive SL, and KARRIKIN INSENSITIVE 2 (KAI2), which mediates KAR signaling in parallel with SL pathways [25,26]. The F-box protein MAX2 and the F-box/Kelch-repeat protein SKP1-INTERACTING PARTNER 25 (SKIP25) were also detected, both belonging to the SCF-type ubiquitin ligase machinery implicated in SL/KAR-dependent protein turnover [9]. In addition, MAX1-like cytochrome P450s (CYP711A) and the carotenoid cleavage dioxygenases CAROTENOID CLEAVAGE DIOXYGENASE 7 (CCD7) and CCD8—enzymes catalyzing key steps in SL biosynthesis from carotenoid precursors—were identified as putative partners [27]. The network further contained RADIALIS-like transcription factors RL2 and RL5, members of the SANT/MYB family that have been implicated in transcriptional regulation and developmental processes and are themselves predicted to associate with CCD7/CCD8 and SMAX1 in *Arabidopsis* [28].

Consistent with this interaction landscape, GO enrichment analysis showed that LaSMXL-interacting proteins were predominantly associated with karrikin and strigolactone response and biosynthesis (GO:0080167, GO:1902347, GO:1901601), protein quality control of misfolded proteins (GO:0006515), shoot development and morphogenesis (GO:0010346, GO:0010223, GO:0001763), and chloroplast organization (GO:0009658) (Table S3).

### 2.7. WGCNA

**Figure 8:**
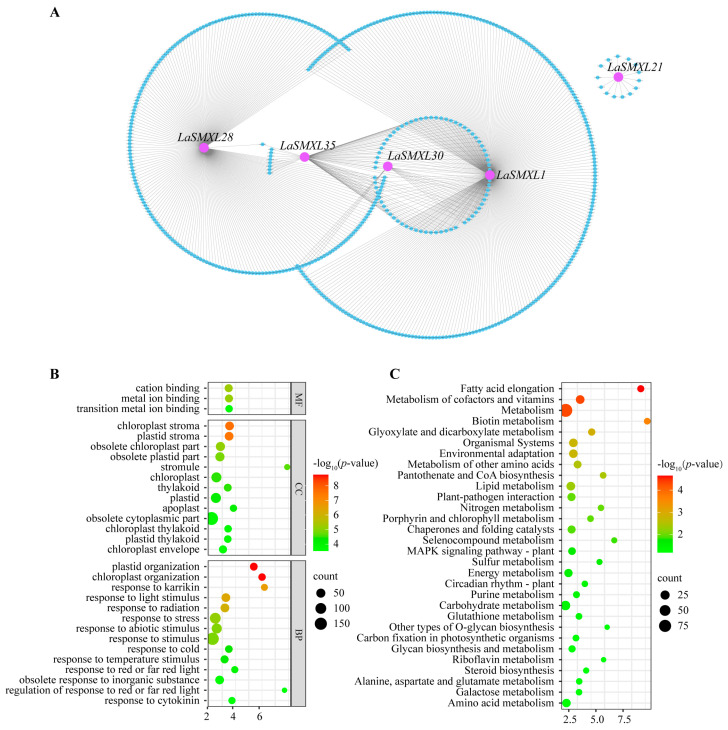
Co-expression networks of *LaSMXL* genes at the transcriptome level. (**A**) Local gene co-expression networks of *LaSMXL* genes. Purple circles represent *LaSMXL* genes, and blue circles represent co-expressed genes. (**B**) GO enrichment analysis of genes co-expressed with *LaSMXL* genes. (**C**) KEGG enrichment analysis of genes co-expressed with *LaSMXL* genes.

To identify *LaSMXL* family members potentially involved in photomorphogenesis, we performed weighted gene co-expression network analysis (WGCNA) on RNA-seq data from multiple lavender tissues. A scale-free co-expression network was constructed, and dynamic tree cutting based on topological overlap dissimilarity grouped genes with similar expression patterns into 14 distinct modules (Figure S3), each representing a putative co-regulated gene set. Among these, the turquoise module contained the largest number of *LaSMXL* genes: this module comprised 2692 genes in total, including five members (*LaSMXL1*, *LaSMXL21*, *LaSMXL28*, *LaSMXL30*, and *LaSMXL35*) (Table S4). Module–trait relationship analysis indicated that the turquoise module showed a moderate positive association with leaf tissue (Pearson’s r = 0.66). To further characterize these five *LaSMXL* genes, we constructed gene-centered co-expression networks (Figure 8A). *LaSMXL1*, *LaSMXL21*, *LaSMXL28*, *LaSMXL30*, and *LaSMXL35* were co-expressed with 120, 130, 10, 150, and 160 genes, respectively. Functional annotation and GO enrichment of these co-expressed genes indicated predominant involvement in chloroplast and plastid development, responses to karrikin and light stimulus (Figure 8B). KEGG enrichment analysis further revealed significant over-representation of metabolic pathways, including fatty acid elongation, metabolism of cofactors and vitamins, biotin metabolism, and glyoxylate and dicarboxylate metabolism (Figure 8C). Taken together, these co-expression patterns suggest that *LaSMXL1*, *LaSMXL21*, *LaSMXL28*, *LaSMXL30*, and *LaSMXL35* are closely associated with light-responsive and chloroplast-related processes in lavender, consistent with roles in photomorphogenesis analogous to those reported for *AtSMXL2* in *Arabidopsis*.

## 3. Discussion

### 3.1. Evolution of the SMXL Gene Family in Lavender

With the rapid accumulation of plant genome sequences, *SMXL* gene families have been systematically identified in an increasing number of species, including *Arabidopsis*, common bean, soybean and poplar, where 8, 9, 31, and 12 members, respectively, have been reported [29,30,31,32]. Comparative phylogenomic analyses across land plants indicate that *SMXL* genes originated early during terrestrial plant evolution and subsequently underwent repeated rounds of duplication and loss, giving rise to four major clades with distinct evolutionary rates and functional properties [33,34]. These studies collectively highlight gene duplication—particularly whole-genome duplication (WGD) and whole-genome triplication (WGT)—as a major driving force in the expansion and diversification of the *SMXL* family.

Against this backdrop, our work extends *SMXL* evolutionary analyses to *L*. *angustifolia*, a perennial aromatic shrub with a relatively large and complex genome. We identified 37 *SMXL* genes in lavender—substantially more than in *Arabidopsis* (8 *SMXL* genes) and rice (6 *SMXL* genes)—indicating a pronounced expansion of the *SMXL* family in this species. Duplication-type classification revealed that none of the *LaSMXL* genes originated from tandem duplication; instead, all copies could be traced back to WGD- or large-scale segmental duplication events, suggesting that genome duplication rather than local tandem amplification was the primary mechanism driving *SMXL* family expansion in lavender.

To place lavender within a broader angiosperm framework, we examined inter-species synteny among lavender, *Arabidopsis* and rice. Lavender exhibited higher collinearity and more syntenic *SMXL* gene pairs with *Arabidopsis* than with rice, consistent with their closer phylogenetic relationship as dicot species. In contrast, the reduced collinearity between lavender and monocot rice likely reflects greater evolutionary divergence and indicates that lineage-specific *SMXL* gene loss and gain have accompanied angiosperm diversification [35].

Evolutionary rate analyses further showed that almost all duplicated *LaSMXL* gene pairs have *K*a/*K*s < 1, indicating that they have been maintained under strong purifying selection following WGD-mediated expansion. Only the *LaSMXL1*/*LaSMXL35* pair displayed a *K*a/*K*s ratio > 1, suggesting that this pair may have experienced positive selection and acquired partial functional innovation. Overall, these findings support a model in which WGD-driven expansion, followed by prolonged purifying selection and limited positive selection, has generated a relatively large yet structurally conserved *SMXL* gene family in lavender while preserving key components of the SL/KAR signaling framework.

### 3.2. Structural Characteristics and Diversification of LaSMXL Genes

Previous work has shown that SMXL proteins in *Arabidopsis* can be classified into four subfamilies—SMAX1/SMXL2, SMXL3, SMXL4/SMXL5, and SMXL6/SMXL7/SMXL8—representing distinct functional branches of the family [29]. By integrating SMXL proteins from lavender, *Arabidopsis*, maize, moss, cacao, rice, and grape into a unified phylogenetic framework, we likewise divided LaSMXL proteins into four major groups that broadly correspond to these *Arabidopsis* clades. Within this framework, lavender Group III consisted almost exclusively of LaSMXL members together with a single cacao protein, forming an independent branch within eudicots. This pattern suggests either lineage-specific expansion in lavender and cacao or preferential retention of this SMXL lineage in certain dicot species. In contrast, several SMXL proteins from *P. patens* formed a distinct cluster at a relatively basal position, consistent with early divergence and/or species-specific retention of *SMXL* copies in bryophytes. Together, these results highlight the coexistence of a conserved evolutionary backbone and lineage-specific remodeling of the *SMXL* family across plant lineages.

Gene structure and conserved motif analyses provided additional support for the phylogenetic groupings observed above. Within each *LaSMXL* subgroup, members shared highly similar exon–intron organizations and motif compositions, indicating strong structural conservation at the subfamily level. Most *LaSMXL* genes possessed both 5′ and 3′ untranslated regions (UTRs), and Group I and Group III genes typically consisted of three exons and two introns, closely resembling the canonical *SMXL* architecture reported in *Arabidopsis*, cotton, and pear [29,36,37]. Group II genes exhibited a broadly similar organization, although the presence of two-exon members and the split-second exon observed in *LaSMXL22* point to modest structural remodeling within this clade. In contrast, Group IV *LaSMXL* genes displayed markedly longer and more complex gene structures, with exon numbers exceeding nine in most cases. This multi-exon architecture resembles that reported for *SMXL* genes in soybean and may reflect subfunctionalization or neofunctionalization driven by intragenic duplication and exon rearrangement, ultimately contributing to functional diversification within the family [31,38].

At the protein level, predicted three-dimensional structures further supported the patterns of conservation and diversification inferred from gene architecture. AlphaFold 3-based modeling indicated that highly expressed LaSMXL proteins in Group I share similar overall folds with higher-confidence predictions, consistent with their conserved exon–intron organization and motif composition (Figure 2D). By contrast, LaSMXL proteins phylogenetically related to AtSMXL1/2 exhibited more variable and lower-confidence predicted conformations, in line with their proposed functional complexity in karrikin signaling. These structural features indicate that coordinated structural changes in both genes and proteins drive the functional divergence among *LaSMXL* subfamilies in lavender.

### 3.3. Expression Patterns and Functional Integration of LaSMXL Genes in Lavender

Gene family expansion is often followed by functional divergence among paralogs, which can manifest as loss of function, partial redundancy or acquisition of novel or partitioned roles [39]. In *L. angustifolia*, the majority of *LaSMXL* genes exhibited very low transcript levels across the tissues examined, whereas only nine members showed high or moderate expression in at least one organ. This expression landscape is consistent with a scenario in which, following WGD-driven expansion, only a subset of *LaSMXL* genes has retained major regulatory functions, while many paralogs may act as conditionally expressed or functionally attenuated copies. Integration of phylogenetic placement with expression profiles provides initial hypotheses regarding *LaSMXL* functions. In *Arabidopsis*, Group I members (*SMAX1*/*SMXL2*) act in the karrikin (KAR) signaling pathway to promote seed germination and hypocotyl elongation and function downstream of KAI2 to regulate root and root-hair development and several abiotic stress responses [29]. Group IV members (*SMXL6*/*7*/*8* and rice *D53*) serve as central repressors of the strigolactone (SL) signaling pathway; their proteasome-mediated degradation upon SL perception suppresses axillary branching and tillering, thereby modulating plant architecture [8,35]. Group III members (*SMXL3*/*4*/*5*) display distinct properties, being largely independent of MAX2-mediated degradation and not directly involved in canonical SL/KAR signaling [11,24]. In lavender, *LaSMXL3*, *LaSMXL19* and *LaSMXL29* cluster with *Arabidopsis SMAX1*/*SMXL2*, suggesting that they may participate in analogous germination- and growth-related signaling processes. By contrast, clear one-to-one orthologs of *Arabidopsis SMXL6*/*7*/*8* or *SMXL3*/*4*/*5* were not detected, indicating substantial remodeling of *SMXL* functional modules after divergence of the lavender and *Arabidopsis* lineages.

Tissue-specific expression analyses further revealed distinct expression clusters among the highly expressed *LaSMXL* genes, consistent with functional sub-specialization. *LaSMXL21*, *LaSMXL1, LaSMXL19*, and *LaSMXL35* formed one cluster with predominant expression in leaves, whereas *LaSMXL30*, *LaSMXL15* and *LaSMXL28* formed a second cluster characterized by broadly high expression in stems, leaves and petals. Within this latter cluster, *LaSMXL28* accumulated to particularly high levels in stems and leaves, suggesting that it may act as a major regulator of aerial organ development, with *LaSMXL30* and *LaSMXL15* providing partial functional redundancy. *LaSMXL29* and *LaSMXL3* exhibited similar expression patterns enriched in floral tissues, while *LaSMXL19* displayed a unique expression profile distinct from all other *LaSMXL* members, implying a potentially specialized role in lavender growth or reproductive development. Notably, *LaSMXL22*, *LaSMXL15*, and *LaSMXL28* maintained consistently high expression throughout bud stage I, bud stage II, and 50% flowering, suggesting roles in sustained floral developmental processes. In contrast, *LaSMXL20*, *LaSMXL36*, and *LaSMXL34* were preferentially expressed at early bud stages but declined at flowering, indicating potential involvement in early organ initiation and progression. The intermediate group with relatively stable but moderate expression (*LaSMXL17*, *LaSMXL27*, *LaSMXL30*, and *LaSMXL14*) may contribute to maintaining developmental competence. Overall, these patterns support functional specialization of *LaSMXL* genes across different phases of floral organ development.

In addition to their putative developmental roles, several *SMXL* genes in other species have been implicated in abiotic stress responses [40]. Consistent with this, transcriptome profiling under different temperature regimes showed that most *LaSMXL* genes exhibited only minimal responsiveness to temperature fluctuations, but a subset of members displayed consistent and monotonic responses to cold exposure. *LaSMXL28* and *LaSMXL14* displayed progressively increased transcript levels as temperature decreased from 20 °C to 0 °C, whereas *LaSMXL33*, *LaSMXL27*, and *LaSMXL17* showed gradually reduced expression across the same gradient. *LaSMXL15* and *LaSMXL30* maintained relatively stable expression under all temperature treatments. Promoter analyses revealed abundant *cis*-acting elements associated with abscisic acid and other phytohormones, together with multiple stress-related motifs, including those linked to drought, low temperature and anaerobic conditions. Collectively, these observations suggest that specific *LaSMXL* members contribute to the transcriptional reprogramming of lavender under abiotic stress, with *LaSMXL28* and *LaSMXL17* emerging as plausible candidates for mediating low-temperature responses.

At the network level, SMXL proteins function as key nodes in SL and KAR signaling by interacting with hormone receptors, SCF-type ubiquitin ligases and downstream transcriptional regulators [41]. Protein–protein interaction predictions for nine highly expressed LaSMXL proteins revealed a network that includes multiple canonical components of SL and KAR biosynthesis and signaling. These comprise the α/β-hydrolase receptors D14 and DAD2, which perceive SLs, and KAI2, which mediates KAR signaling in parallel [26,42]. The F-box protein MAX2 and the F-box/kelch-repeat protein SKIP25 (KUF1) were also recovered, both forming part of SCF-type ubiquitin ligase complexes implicated in SL/KAR-dependent protein degradation [43,44]. In addition, MAX1-like cytochrome P450s (CYP711A) and the carotenoid cleavage dioxygenases CCD7 and CCD8—enzymes catalyzing key steps in SL biosynthesis—were identified as putative partners, together with RADIALIS-like transcription factors RL2 and RL5, SANT/MYB-type regulators associated with developmental control and predicted to interact with CCD7/CCD8 and SMAX1 in *Arabidopsis* [27,45]. Consistent with this interaction landscape, GO enrichment analysis showed that LaSMXL-interacting proteins were significantly associated with karrikin and strigolactone response and biosynthesis, protein quality control of misfolded proteins, shoot development and morphogenesis, and chloroplast organization. These findings indicate that LaSMXL proteins are embedded in a conserved SL/KAR signaling framework that links hormone biosynthesis, perception and protein turnover to developmental outputs and chloroplast function. Notably, the genetic transformation and functional validation system for lavender is still under development; therefore, the functional roles inferred in this study should be considered predictive. Nevertheless, functional validation of *SMXL* genes has been successfully performed in several model or related plant species, providing useful precedents for lavender. Future studies will aim to experimentally verify the predicted interactions and potential functions of *LaSMXL* genes using approaches such as genetic transformation, CRISPR/Cas9-mediated gene editing, and yeast two-hybrid (Y2H) assays.

To complement the PPI analysis, we integrated *LaSMXL* genes into a genome-wide co-expression network using weighted gene co-expression network analysis. The module containing the largest number of *LaSMXL* genes was strongly associated with leaf tissue and enriched in genes involved in chloroplast and plastid development, responses to karrikin and light stimuli, and core metabolic pathways such as fatty acid elongation, cofactor and vitamin metabolism, biotin metabolism and glyoxylate/dicarboxylate metabolism. Local co-expression sub-networks centered on *LaSMXL1*, *LaSMXL21*, *LaSMXL28*, *LaSMXL30*, and *LaSMXL35* consistently pointed to roles in light perception, plastid biogenesis and chloroplast-associated metabolism. These network properties, together with promoter architecture and tissue-specific expression patterns, support a model in which a subset of *LaSMXL* genes is tightly integrated into hormone-, light- and plastid-related signaling networks and contributes to photomorphogenesis and chloroplast function in lavender [29,41]. Taken together, the combined expression, stress-response, PPI, and co-expression evidence indicate that *LaSMXL* genes do not simply recapitulate the functional partitioning described in *Arabidopsis*, but instead constitute a rewired and partially diversified signaling module. While some *LaSMXL* members likely retain conserved roles analogous to SMAX1/SMXL2, others appear to have acquired lavender-specific functions at the intersection of developmental regulation, hormone signaling and environmental adaptation. Therefore, these proposed functions should be regarded as hypotheses pending direct genetic and molecular validation in lavender.

## 4. Materials and Methods

### 4.1. Plant Materials

The plant materials used in this study were collected from lavender cultivars grown under natural open-field conditions at the resource nursery of the Institute of Agricultural Sciences, Fourth Division of the Xinjiang Production and Construction Corps (Yili, Xinjiang, China). Petals and sepals from mature flowers of *L*. *angustifolia* at different developmental stages (bud stage, flowering stage, withering stage) were collected. Additionally, root, stem, and leaf tissues from 2-month-old plants were harvested. All samples were flash-frozen in liquid nitrogen and stored at −80 °C until RNA extraction.

### 4.2. Identification of the SMXL Gene Family

Protein sequences of eight *Arabidopsis SMXL* family members were downloaded from the TAIR database (http://www.arabidopsis.org/) (accessed on 19 April 2025), and the lavender genome data was obtained from the NCBI database (https://www.ncbi.nlm.nih.gov/) (accessed on 10 September 2024) [46,47]. A local BLASTP search was conducted using the *Arabidopsis* SMXL protein sequences as queries, with an e-value threshold of less than 1 × 10^−10^. Simultaneously, multiple sequence alignment of the Arabidopsis SMXL proteins was performed, and an HMM model was built for further analysis using hmmbuild (from HMMER v3.4). Both local BLAST+ (v2.14.0) and hmmsearch analyses were conducted using TBtools-II (v2.102) [48]. Results from both searches were integrated, and the sequences were submitted to the Pfam database for domain identification. Lavender SMXL family members were identified based on the presence of Clp-N and P-loop ATPase domains. Physical properties of the identified proteins, including protein length, molecular weight (MW), isoelectric point (pI), instability index (II), and grand average of hydropathicity (GRAVY), were calculated using the ExPasy server (https://web.expasy.org/protparam/) (accessed on 24 April 2025). Chromosomal positions of *LaSMXL* genes were obtained from the genome annotation and visualized using TBtools.

### 4.3. Phylogenetic and Genetic Structure Analysis

SMXL protein sequences from *A. thaliana*, *O. sativa*, *Z. mays*, *V. vinifera*, *P. patens*, and *T. cacao* were downloaded from the NCBI database (https://www.ncbi.nlm.nih.gov/) (accessed on 22 April 2025). The sequences were aligned using ClustalW (v2.1) with default parameters [49]. A Neighbor-Joining phylogenetic tree was constructed using MEGA 7.0 with 1000 bootstrap replicates, and the resulting tree was visualized using Evolview (http://www.evolgenius.info/evolview/) (accessed on 3 September 2025) [50,51]. Gene structure annotations for *LaSMXL* genes were obtained from the lavender genome annotation data, and the intron-exon structure of the *LaSMXL* genes was visualized using TBtools. Conserved protein motifs were identified using MEME (https://meme-suite.org/tools/meme) (accessed on 22 April 2025) with a maximum of 10 motifs. The 3D structures of LaSMXL proteins were predicted using AlphaFold (https://alphafold.com/) (accessed on 25 November 2025).

### 4.4. Genome Synteny and Ka/Ks Ratios

Genome collinearity within the lavender genome and between lavender and other species was analyzed using MCScanX (2012), with an e-value cutoff of 1.0 × 10^−5^. Collinear gene pairs were considered duplicated genes for further analysis [52]. The non-synonymous (*K*a) and synonymous (*K*s) substitution rates for each pair of duplicated *LaSMXL* genes were calculated using KaKs_Calculator [53]. Selection pressure on these genes was assessed by calculating the *K*a/*K*s ratio, with *K*a/*K*s > 1, =1, and <1 indicating positive selection, neutral selection, and negative selection, respectively.

### 4.5. Analysis of Promoter Cis-Elements

The 2000 bp upstream regions of all *LaSMXL* genes were extracted using TBtools and analyzed using the PlantCARE tool (http://bioinformatics.psb.ugent.be/webtools/plantcare/html/) (accessed on 25 April 2025). Statistical analysis of the number and types of *cis*-acting elements in the promoter regions was performed using Excel and visualized as a heatmap using the R package (ggplot2 v3.5.0) [54].

### 4.6. Expression Analysis

The raw transcriptome data for *L*. *angustifolia*, including flower buds, sepals at flowering and withering stages, petals, leaves, and stems from 2-month-old plants, as well as cold-stressed samples, were obtained from NCBI (Project ID: PRJNA892961). Transcriptome data for floral organs from two lavender cultivars (*Xinxun 2* and *YXA-5*) across three developmental stages were obtained from NCBI (SRP158322). Developmental stages were defined using spike length and the proportion of open flowers: Bud I, spike length ≤0.5 cm; Bud II, spike length 0.5–1.0 cm; 50% flowering, the stage at which 50% of flowers on each spike were open. Raw sequences were assessed for quality using fastp 0.24.0 [55], and low-quality reads were filtered using Trimmomatic v0.39 [56]. Cleaned reads were aligned to the lavender reference genome using HISAT2 v2.2.1 [57]. Gene expression was quantified with featureCounts v2.0.3, and expression levels were normalized using log_2_(FPKM + 1). Heatmaps of gene expression patterns were generated using TBtools.

For quantitative PCR (qPCR), RNA was reverse transcribed using 1 μg of total RNA and HiScript III All-in-one RT SuperMix for qPCR. qPCR was performed using SYBR Premix Ex Taq™ II (Takara, Kusatsu, Japan) on a Bio-Rad CFX96 Touch™ system (Bio-Rad, Hercules, USA). Lavender *Actin* gene was used as an internal control for normalization. Relative transcript levels were calculated using the 2^−ΔΔCt^ method [58]. The experiment included three independent biological replicates, each with three technical replicates. Primer sequences are provided in Supplementary Table S5.

### 4.7. Prediction of Protein–Protein Interaction (PPI) Network

PPI networks for highly expressed *LaSMXL* genes were predicted using the STRING database (https://string-db.org/) (accessed on 8 May 2025). Protein sequences of these genes were submitted to the STRING platform to predict potential interacting partners. Interaction data were integrated and visualized using Cytoscape software (v3.10.2) for further analysis [59].

### 4.8. WGCNA

WGCNA was performed on transcriptomic data from various lavender tissues using the WGCNA platform integrated into TBtools. The gene expression matrix was filtered to retain the top 5000 highly variable genes with the highest median absolute deviation (MAD) across all samples. Outlier samples with abnormal expression profiles were removed using clustering analysis. The minimum soft thresholding power (β) was selected to ensure a scale-free topology fit index > 0.85, and a weighted adjacency matrix was constructed. This matrix was transformed into a topological overlap matrix (TOM), and hierarchical clustering was performed based on TOM dissimilarity (1 − TOM). Gene co-expression modules were identified using the dynamic tree cut algorithm, and modules with eigengene correlations > 0.75 were merged. Correlations between module eigengenes and external tissue traits were computed for statistical significance. The modules most enriched in *LaSMXL* genes were selected for further analysis, including functional enrichment and KEGG enrichment. The analysis was performed using the R package clusterProfiler, and bubble plots were generated for visualization [60].

## 5. Conclusions

In summary, this study presents the first genome-wide identification and comprehensive analysis of the *SMXL* gene family in Lavandula angustifolia. We identified 37 *LaSMXL* genes, which phylogenetically cluster into four distinct subfamilies. The results indicate that this family has predominantly expanded through whole-genome and segmental duplication events, undergoing structural and functional diversification under strong purifying selection. Integrated analyses of transcriptomic profiles, protein–protein interactions, and co-expression networks further revealed that a subset of *LaSMXL* members exhibits pronounced developmental stage specificity and low-temperature responsiveness. Furthermore, these highly expressed members are intimately associated with conserved strigolactone/karrikin (SL/KAR) signaling pathways and chloroplast functions. Collectively, these findings broaden our understanding of the evolution, developmental regulation, and cold-stress responses of the *SMXL* gene family in aromatic plants. Importantly, this work provides key candidate regulatory genes for future functional validation, laying a theoretical foundation for molecular breeding strategies aimed at optimizing plant architecture, enhancing inflorescence-derived essential oil yield, and improving climate resilience in lavender.

## Data Availability

The transcriptome datasets analyzed in this study are publicly available in the NCBI BioProject database under accession numbers PRJNA892961 (tissue-specific data of *L. angustifolia* ‘*Xinxun 1*’), SRP158322 (Transcriptome data for floral organs from ‘*Xinxun 2*’ and ‘*YXA-5*’) and PRJNA765132 (cold stress-related data). All other data supporting the findings of this study are included in the article and Supplementary Materials.

## Supplementary Materials

The following supporting information can be downloaded at: https://www.mdpi.com/article/10.3390/ijms27104461/s1.
