# Supplementary material for: Genome-Wide Identification and Characterization of the SMXL Gene Family in Lavandula angustifolia
Source: Int J Mol Sci. 2026 May 16;27(10):4461. doi: 10.3390/ijms27104461 (PMC13207650; doi:10.3390/ijms27104461)
Supplement: Supplementary file 1 [file ijms-27-04461-s001.zip › supplementary file 1.pdf]

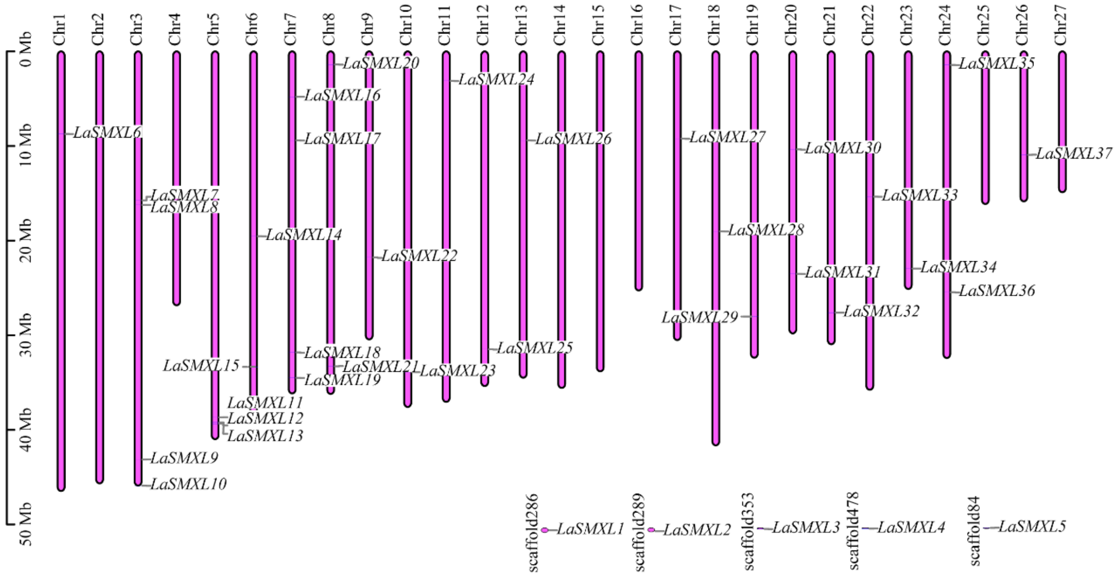

Figure S1. Chromosomal distribution of *LaSMXL* genes in *Lavandula angustifolia*. Thirty-seven *LaSMXL* genes are unevenly distributed across 20 of the 27 chromosomes, while the remaining genes are located on unanchored scaffolds.

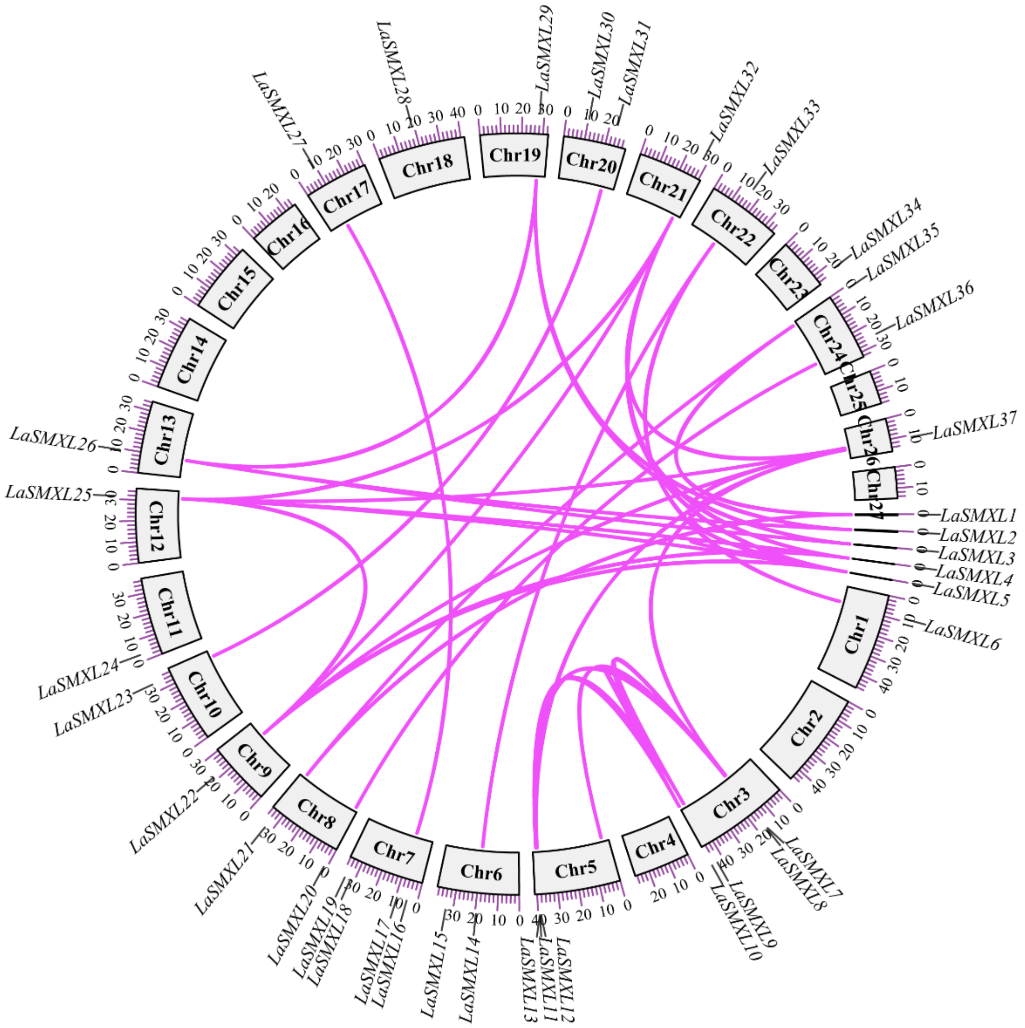

Figure S2. Intragenomic syntenic relationships of *LaSMXL* genes in *Lavandula angustifolia*.

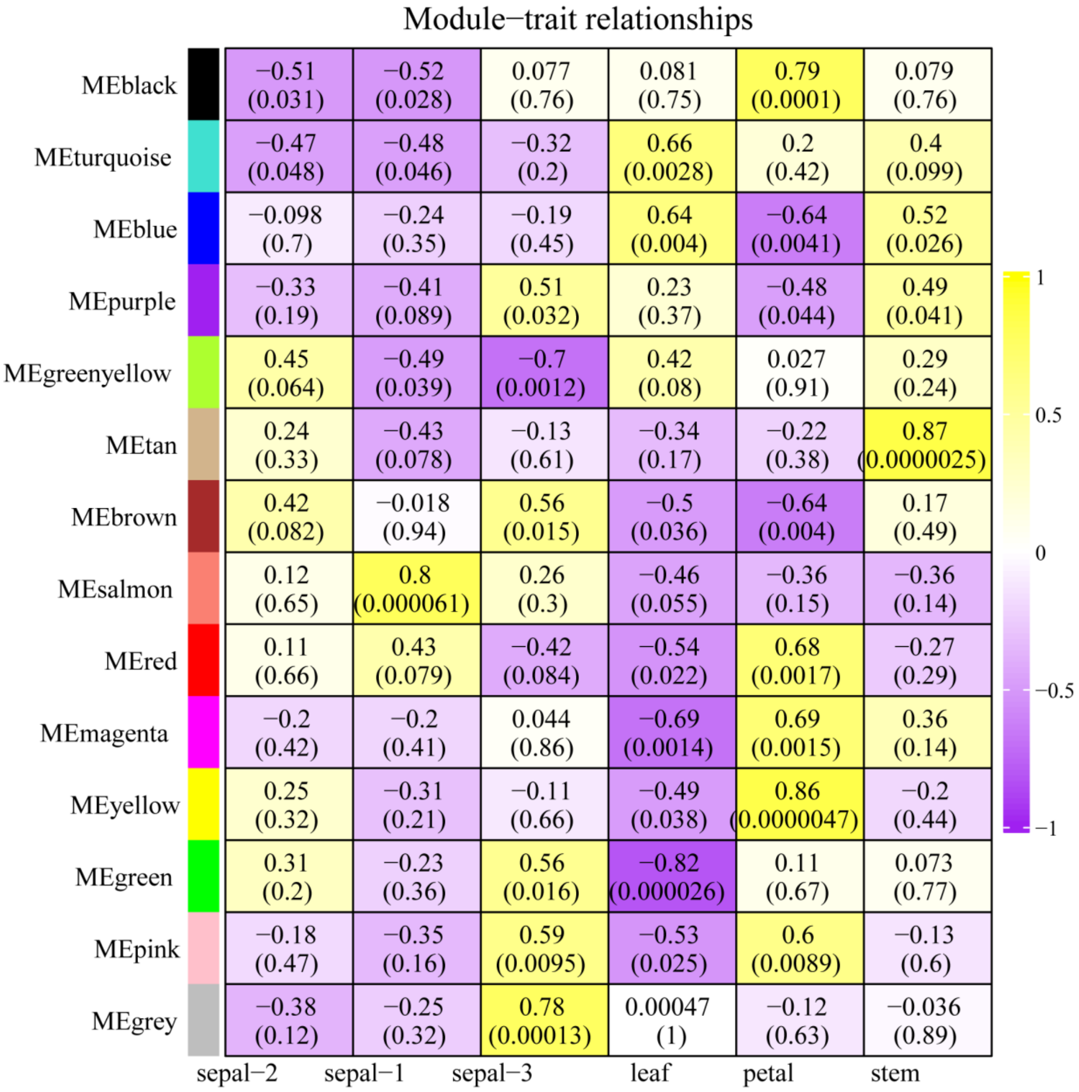

Figure S3. Module–trait relationships identified by weighted gene co-expression network analysis (WGCNA). Heatmap showing correlations between gene co-expression modules and tissue traits in *Lavandula angustifolia*.
